# Assessing the economic impact of catheter-related bloodstream infections when switching from open to closed system delivery of human albumin solutions in ICUs

**DOI:** 10.1186/cc12314

**Published:** 2013-03-19

**Authors:** F Axelsen, P Riss

**Affiliations:** 1 Baxter Healthcare, Zurich, Switzerland; 2 Medical University of Vienna, Austria

## Introduction

Hospital-acquired infections create a significant burden to the healthcare system. To understand the economic impact, a model was developed to assess the cost of differences in catheter-related bloodstream infections (BSI) in ICUs based on either closed or open albumin systems.

## Methods

A model was developed with the aim of showing the economic consequences of differences between intravenous (i.v.) infusions of albumin via open or closed infusion containers. The model took a healthcare perspective in the UK. The impact of central venous catheter-related BSI was evaluated. The model took differences in BSI rates and the associated cost consequences for the hospital into account. The model bases on the evidence that closed system delivery helps to reduce the risk of external contamination, which decreases the BSI rate and reduces overall mortality [1-3]. We assessed the total costs using public list prices and literature. The model assumes that infection rates for using open and closed albumin delivery systems are similar to any other closed or open nonalbumin i.v. fluid delivery system used in the ICU. The model looks at reduction in central-line-associated BSI and the potential cost saving of this reduction.

## Results

The economic model shows the potential cost saving by switching from open to closed albumin administration systems. It is measured in occurrence of BSI in the ICU, estimated occurrence of BSI in the albumin population and incidence of overall mortality in the ICU and cost of BSI per albumin patient. Finally, the model describes the potential cost savings by switching to a closed system. Using closed albumin delivery systems may overall provide a cost saving for the hospital and healthcare sector. Assuming 800 patients treated in an ICU per year with all in all 4,800 ml albumin, the total annual cost saving would be £24,000 switching from open to closed albumin infusions.

## Conclusion

Switching from an open to a closed albumin infusion system may result in a reduction in the risk of external contamination, resulting in less infection and may provide a cost saving for the healthcare system.
